# Monthly Summary

**Published:** 1880-09

**Authors:** 


### MONTHLY SUMMARY.

*Chancres of the Tonsil and Mouth.*—M. Spillman reports, in the *Revue Medicale de l' Est*, two cases remarkable for the difficulty in the diagnosis of one of them, and especially because of the conditions under which contagion took place :

A lady, fifty-nine years of age, placed by her position beyond all the ordinary causes of syphilitic contagion. presented herself for examination, complaining of a slight sore throat dating back more than a fortnight. There were sharp pains felt at the moment of swallowing, and a considerable glandular swelling situated at the angle of the jaw on the right side. Upon examining the throat there was observed upon the tonsil of the same side an ulcer the size of a three-cent piece, superficial, slightly excavated, and covered with a grayish secretion. The mucus membrane around this ulceration was œdematous and formed like a cushion. No other lesion was found in the buccal mucous membrane or in the isthmus of the fauces. The ganglia of the perotid region were voluminous and painful on pressure. No redness of the skin existed, however. With this exception, the patient complained of no trouble in her general health, and seemed to attach but little importance to the disease from which she suffered. Knowing this patient's manner of life, M. Spillman was unable to suspect syphilis, nevertheless at the end of some days a roseola made its appearance, which permitted no doubt as to the existence of syphilis of which the ulcer in the throat was the primitive lesion. The difficult question of the etiology remained. After much research it was learned that the patient, having received into her house a child raised on the bottle, had the habit of often aspirating the instrument to ascertain the temperature of the milk, by means of the rubber mouth-piece. Now, it was learned that the child thus nourished was affected with hereditary syphilis with multiple lesions of the mouth and genital parts.

A second case reported by M. Spillman is not less interesting in relation to the mode of contagion. This concerned an upholster's apprentice, aged thirteen years, who for several days had borne upon the right side and anterior surface of the lower lip a small red patch, the size of a three-cent piece. This patch was indurated at its base, and the ganglia were enlarged; in fact, it was a labial chancre. Nevertheless, it appeared impossible to find the source of infection until it was learned that this child worked with a workman and took his tacks from the same sack with him. Now, upholsters have a habit of putting a small handful of tacks into their mouths, which they make use of in proportion to the requirements of their work, and put the surplus tacks back into the sack. Upon examination, this patient presented numerous mucous patches about the mouth: hence it is more than probable that the child became infected through placing in his mouth tacks impregnated with this man's saliva.—*St. Louis Clinical Record.*

---

*Effects of Smoking on the Heart.*—Some years ago M. Decaisne drew attention to the fact that tobacco-smoking often causes an intermittent pulse. Out of eighty-one great smokers examined, twenty-three presented an intermittent pulse, independent of any cardiac lesion. This intermittency disappeared when the habit of smoking was abandoned. He also studid the effects of smoking on children from nine to fifteen years of age, and found that it undoubtedly caused palpitation, intermittent pulse, and chloro-anæmia. The children, furthermore, became dull, lazy, and predisposed to the use of alcoholic drinks. Recently he reported to the *Societe d' hygiene* the results of his observations on the effects of smoking on women. Since 1865 he has met with and observed forty-three female smokers. Most of them suffered from disturbances of menstruation and digestion, and eight presented very marked intermittency of the pulse without any lesion of the heart. He gave detailed accounts of these eight cases, in which all treatment directed against the intermittency proved utterly useless, while the suppression of tobacco was invariably followed by improvement and very often by complete disappearance of the phenomenon.—*Gazette Obstetricale.*

